# Treatment of Hepatitis C in Children: A Systematic Review

**DOI:** 10.1371/journal.pone.0011542

**Published:** 2010-07-13

**Authors:** Jia Hu, Karen Doucette, Lisa Hartling, Lisa Tjosvold, Joan Robinson

**Affiliations:** 1 Department of Pediatrics, University of Alberta, Edmonton, Canada; 2 Department of Medicine, University of Alberta, Edmonton, Canada; 3 Alberta Research Centre for Health Evidence, University of Alberta, Edmonton, Canada; Sun Yat-Sen University, China

## Abstract

**Background:**

Current guidelines recommend children be treated for hepatitis C virus (HCV) using the same principles applied in adults. There are however few published studies which assess the efficacy and safety of HCV therapy in children.

**Methodology/Principal Findings:**

A systematic review of the literature was completed for studies of any design that evaluated HCV therapy in children. The primary outcome was sustained virologic response (SVR), with sub-group analysis of response rates by genotype. There were 4 randomized controlled trials (RCTs) and 31 non-randomized studies, all involving interferon, pegylated interferon (PEG-IFN), or combinations of these drugs with ribavirin. The SVR rate could not be directly compared as the populations and interventions differed across studies. Genotype was not reported or differed substantially from study to study. The overall SVR rate for PEG-IFN and ribavirin ranged from 30 to 100% which is comparable to the rate in adults. Similar to adults, the SVR rates were significantly higher in children with genotype 2 or 3 compared to genotype 1. Adverse effects were primarily flu-like symptoms and neutropenia. There were insufficient data to assess the applicability of the week 12 stop rule (stopping therapy at week 12 if there is less than a 2 log drop in HCV RNA) or the efficacy of shortening therapy to 24 weeks in children with genotype 2 and 3.

**Conclusions/Significance:**

Current guidelines for the treatment of HCV in children are based on limited data. Further research is needed to define the optimal therapy for HCV in children.

## Introduction

Treatment guidelines for hepatitis C virus (HCV) infection in adults are based on a large body of published natural history studies and randomized controlled trials (RCTs). There is a relative paucity of data regarding the impact of HCV on the morbidity and mortality of infected children and few published studies which assess the efficacy and safety of HCV therapy in children. Despite this, current guidelines recommend children be assessed and treated for HCV in a similar manner to adults [1].

The prevalence of HCV infection in children varies widely by country, ranging from 0% in Japan and 0.4% in Italy to up to 14.5% in Cameroon.[2], [3], [4] A study performed in the early 1990s in the United States documented HCV antibodies in 0.2% of children aged 6 to 12 and 0.4% of children aged 12 to 19 [5]. It is estimated that 75–80% of children who are antibody positive are also HCV RNA positive [5].

The natural history of HCV in children is not completely understood. However, compared to adult infection, spontaneous clearance is more common and both fulminant hepatitis and progression of chronic infection to advanced fibrosis and cirrhosis are less likely [6], [7], [8], [9], [10], [11]. Despite the overall more favorable prognosis than in adults, approximately 4 to 6% of children with chronic HCV infection have evidence of advanced fibrosis or cirrhosis on liver biopsy [11], [12], and 4 to 5 children each year undergo liver transplantation in the United States for end-stage liver disease as a consequence of HCV [13].

Despite the significant number of children infected with HCV, and a clear subset of those who may benefit from HCV therapy, there is little consensus on when or how to optimally treat children with HCV infection. A previous meta-analysis examining interferon monotherapy for pediatric HCV concluded that the sustained virologic response (SVR) was higher and that therapy was better tolerated than in adults [14]. Combination therapy with pegylated interferon (PEG-IFN) and ribavirin has been shown to be superior to standard interferon monotherapy or combination therapy with interferon and ribavirin and is the current gold standard for HCV therapy in adults [1], [15], [16] but there is a paucity of pediatric RCTs of this therapy.

This paper represents a comprehensive and systematic review of evidence specific to children for the treatment of HCV infection. The primary aim was to determine if there is sufficient evidence to recommend any ways in which the approach to therapy of children with HCV should differ from the approach in adults. A secondary aim was to use this data to serve as a basis to identify priority areas for future research.

## Methods

### Searching

A medical research librarian conducted comprehensive searches in the following electronic databases: Medline® (1950-April Week 2 2009), Embase (1980 to 2009 Week 16), EBM Reviews – Cochrane Central Register of Controlled Trials (1^st^ Quarter 2009), Web of Science® (1900–2009), Scopus® (1966–2009), LILACS (1982–2009), Biosis Previews® (1926–2009), Proquest Dissertations and Theses (1900–2009) and the ARIF Reviews Database (1996–2009). No language restrictions were applied. Search strategies were modified to accommodate the controlled vocabulary and search language of each database. The search strategy for Medline appears in Appendix S1. Search strategies for the other databases are available from the corresponding author.

Unpublished studies were identified by hand searching the following conference proceedings: North American and European Societies of Pediatric Gastroenterology and Nutrition (2007–2009), American Association for the Study of Liver Diseases (2006–2009), American Gastroenterology Association (2006–2010), European Association for the Study of the Liver (2007–2010). Ongoing studies were identified through searches of UMIN-CTR, Current Controlled Trials and Clinical Trials.gov. Studies were also located by scanning reference lists of existing systematic reviews and included studies

### Study Selection and Characteristics

The title and abstracts of studies identified by the search were screened for potential relevance. The full text of all potentially relevant studies was reviewed to determine if they fulfilled the eligibility criteria. Studies were included if: 1) they included only children (≤18 years of age) or presented data separately for children; 2) HCV infection was confirmed by detection of HCV RNA; 3) details of the treatment regimen were provided; and 4) data were provided for the sustained virologic response (SVR, defined as a negative HCV RNA at least 24 weeks after cessation of therapy) which was the outcome of interest. All interventions for the treatment of HCV were included, as were all comparisons. We anticipated that there would be a small number of RCTs meeting our inclusion criteria; therefore, we chose to include studies of any design that reported the efficacy or effectiveness of treatment in a cohort of children infected with HCV.

### Validity assessment of randomized controlled trials

All relevant RCTs were assessed for risk of bias independently by two reviewers (JH, LH) using the Cochrane Risk of Bias (RoB) tool. The Cochrane RoB tool evaluates six domains including: sequence generation, allocation concealment, blinding, missing outcome data, selective outcome reporting, and “other sources of bias.” Disagreements were resolved through discussion.

### Data abstraction

An electronic data extraction form was developed in MS Excel a priori and used for data entry. Extracted items included characteristics of the study population and intervention and control groups, inclusion and exclusion criteria, treatments given, SVR, and adverse effects. Data from RCTs was extracted by a single reviewer and checked by a second reviewer. Authors of RCTs with missing data were contacted for details.

### Quantitative data synthesis

There was significant clinical and methodological heterogeneity across studies in terms of the interventions, comparisons, and study designs. Therefore, meta-analysis was not appropriate. We described the studies qualitatively and present detailed results in evidence tables. The results are presented by study design, i.e., RCTs and non-randomized studies with separate analysis for genotype 1 versus other genotypes where specified.

## Results

### Flow of included studies

**Figure 1:**
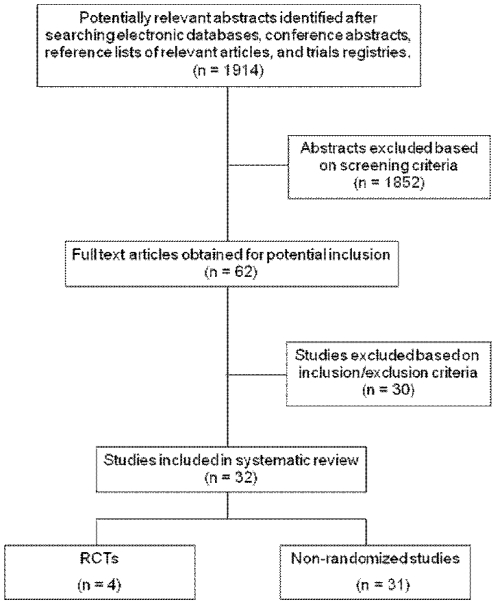
Systematic review process flowchart.

The comprehensive literature search returned a total of 1914 articles (Figure 1). Of these, 4 met the inclusion criteria as RCTs while 31 met the inclusion criteria as non-randomized studies.

### Study Characteristics - Randomized Controlled Trials

Among the four relevant RCTs, three were peer-reviewed manuscripts and one was an abstract presented at the 2008 Annual Meeting of the American Association for the Study of Liver Diseases (Table 1). Three included only pediatric populations [17], [18], [19] while one had a mixed pediatric-adult population [20]. All four RCTs were deemed to have an unclear risk of bias based on the Cochrane Risk of Bias tool. The RCT by Schwarz et al. [19] appeared to be the most methodologically rigorous but was still rated unclear since only the study protocol and abstract were available for assessment. Sequence generation and allocation concealment were generally poorly described. Inappropriate sequence generation and inadequate allocation concealment can result in overestimates of treatment effects.

**Table 1:** Study design of randomized controlled trials of pediatric HCV therapy.

Author/Year/Country	Entry criteria	Group A Characteristics	Group B Characteristics	Group A Treatment	Group B Treatment
1. Iorio 1995, Italy [18]	Children with HCV attending liver diseases clinic 1 to 14 years of age with chronic hepatitis on biopsy, elevated ALT for at least 6 months, and anti-HCV antibodies	7 males, 4 females;7 genotype 1, 5 non-genotype 1	4 males, 6 females;7 genotype 1, 3 non-genotype 1	Interferon alfa, 3 MU/m^2^times weekly for 12 months	No treatment
2. Bortolotti, 1995, Italy [17]	Children with HCV 2 to 14 years of age with chronic hepatitis on biopsy, elevated ALT for at least 6 months, and anti-HCV antibodies	7 males, 7 females;7 genotype 1, 7 non-genotype 1	5 males, 8 females;6 genotype 1, 8 non-genotype 1	Interferon alfa-2b 5 MU/m^2^ 3 times weekly for 12 months	No treatment
3. Fried 2002, USA [20]	Adolescents with HCV and haemophilia 13 to 17 years of ages and with detectable HCV-RNA	17 adolescents;All infected by transfusion	20 adolescents;All infected by transfusion	Interferon alfa-2b 3 MU/m^2^ 3 times weekly + 1000 mg ribavirin daily for 48 weeks	Interferon alfa-2b 3 MU/m^2^ 3 times weekly for 48 weeks
4. Schwarz 2008, USA [19]	Children with HCV 5 to 17 years of age with chronic hepatitis on biopsy and detectable HCV-RNA	34 males, 21 females;44 genotype 1; 11 non-genotype 1	29 males, 30 females;48 genotype 1; 11 non-genotype 1	PEG-IFN alfa-2a 180 micrograms/1.73 m^2^ + ribavirin 15/mg/kg/day for 48 weeks	PEG-IFN alfa-2a 180 micrograms/1.73 m^2^ for 48 weeks

### Quantitative Data Synthesis - Randomized Controlled Trials

Two studies conducted in Italy were both published in 1995, and randomized children up to 14 years of age with biopsy-proven chronic hepatitis and elevated ALT to interferon (using different regimens in each study as shown in Table 1) versus no treatment for 12 months. Iorio et al. found that 5 of 11 (45%) treated patients and 1 of 11 controls (9%) achieved a SVR [18] (Table 2). These results were borderline statistically significant (p = 0.056), likely due to the small numbers of patients. Bortolotti et al. reported that SVR was achieved in 9 of 13 (69%) treated patients and 0 of 13 controls (p<0.001) [17]. Almost all treated patients had flu-like symptoms, and doses of interferon were decreased in 7 of 25 children (28%) due to neutropenia. Therapy was stopped in 3 other children for ALT flares.

**Table 2:** Outcome of randomized controlled trials of pediatric HCV therapy.

Author/Year/Country	Group A Therapy Incomplete	Group B Therapy Incomplete	Group A SVR	Group B SVR	Group A Adverse Effects	Group B Adverse Effects
1. Iorio 1995, Italy	Discontinued in 1 child in first month from ALT flare-up and in 5 others at 6 months since no response in ALT	Not applicable as no therapy given	5 of 11 (45%) at 30 months	1 of 11 (9%) at 30 months	Transient influenza-like syndrome in all patients;other symptoms included anorexia, asthenia, irritability, headache, abdominal pain, and leukopenia	Not reported
2. Bortolotti, 1995, Italy	Discontinued in 2 children from ALT flare-up, in 1 child from febrile seizure, and 1 since no response in ALT	Not applicable as no therapy given	9 of 13 (69%) at 24 months	0 of 13 (0%) at 24 months	Transient influenza-like syndrome in all patients;Other symptoms included pruritus, weight loss, and leukopenia	Not reported
3. Fried 2002, USA	Not reported	Not reported	10 of 17 (59%) at 72 weeks	Not reported	Not reported	Not reported
4. Schwarz 2008, USA	Discontinued in 2 children	Discontinued in 4 children	29 of 55 (53%) at 72 weeks	15 of 59 (21%) at 72 weeks	Not reported	Not reported

Fried et al. studied a mixed pediatric-adult population at US hemophiliac clinics [20]. Viremic subjects aged 13 years and older were randomized to interferon alfa-2b and ribavirin versus interferon monotherapy for 48 weeks. The only outcome data provided for adolescents was a SVR in 10 of 17 (59%) in those on combination therapy. The authors were contacted and were not able to provide SVR rates in adolescents on monotherapy, but pointed out that only 6 of 57 adults and adolescents combined (11%) achieved SVR on monotherapy.

A fourth RCT by Schwarz et al. randomized children with biopsy-proven chronic hepatitis and viremia in Europe, the US, and South America to PEG-IFN alfa-2a 180ug/1.73m2/week and ribavirin 15 mg/kg/day versus PEG-IFN alfa- 2a monotherapy for 48 weeks [19]. Data was extracted from an abstract published in 2008. SVR was achieved in 29 of 55 (53%) children on combination therapy and in 12 of 59 (21%) on monotherapy (p<0.001). Four percent of children on combination therapy and 7% on monotherapy required early discontinuation, presumably due to adverse events, while 51% on combination therapy and 54% on monotherapy required dose reduction. An abstract from this study described one child with ischemic retinopathy, one with uveitis, and one with transient monocular blindness that the authors attributed to PEG-IFN [21]. The presence of autoantibodies did not alter the SVR rate [22].

Among the four RCTs, viral genotype was only mentioned in the Schwarz study with SVR being 80% in those with non-genotype 1 on combination therapy versus 36% on monotherapy. SVR for those with genotype 1 was 47% on combination therapy versus 18% on monotherapy.

### Study Characteristics - Non-randomized Studies

Thirty-one non-randomized studies met the inclusion criteria (Table S1). Full manuscripts were available for 26 of these studies; the others were only available in abstract form. One study had a comparison group: interferon/ribavarin combination therapy versus interferon monotherapy [23]. The remaining studies were uncontrolled before-after studies; therefore, 32 treatment groups were available for analysis.

The studies were published between 1992 and 2010. The number of participants ranged from 8 to 151 (median: 24). All studies described one of the following four therapies in various standardized doses, most commonly 3 or 5 MU/m^2^ interferon thrice weekly or PEG-IFN alfa-2b 1.0 to 1.5 ug/kg/week and 15 mg/kg/day ribavirin:

1. Interferon monotherapy – 16 treatment groups
2. PEG-IFN monotherapy – 1 treatment group
3. Interferon + ribavarin combination therapy – 6 treatment groups
4. PEG-IFN + ribavarin combination therapy – 9 treatment groups

### Quantitative Data Synthesis - Non-randomized Studies

**Figure 2:**
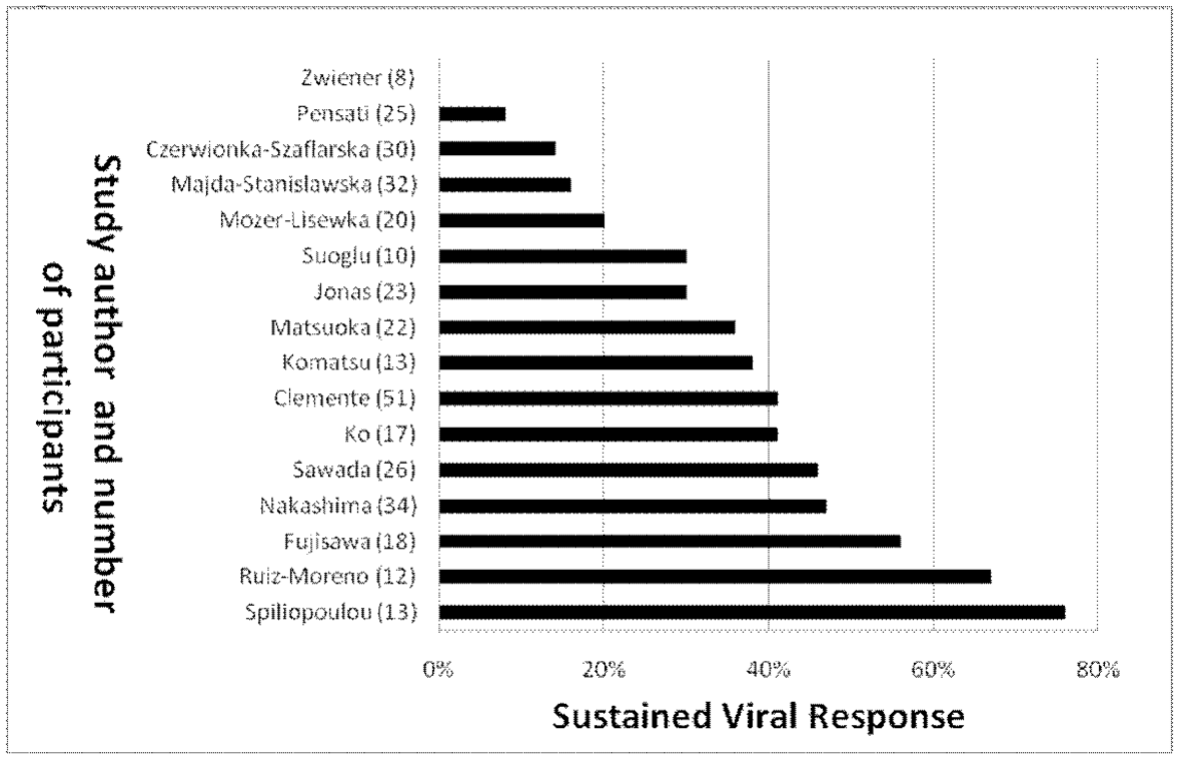
SVR of interferon monotherapy in non-randomized studies.

**Figure 3:**
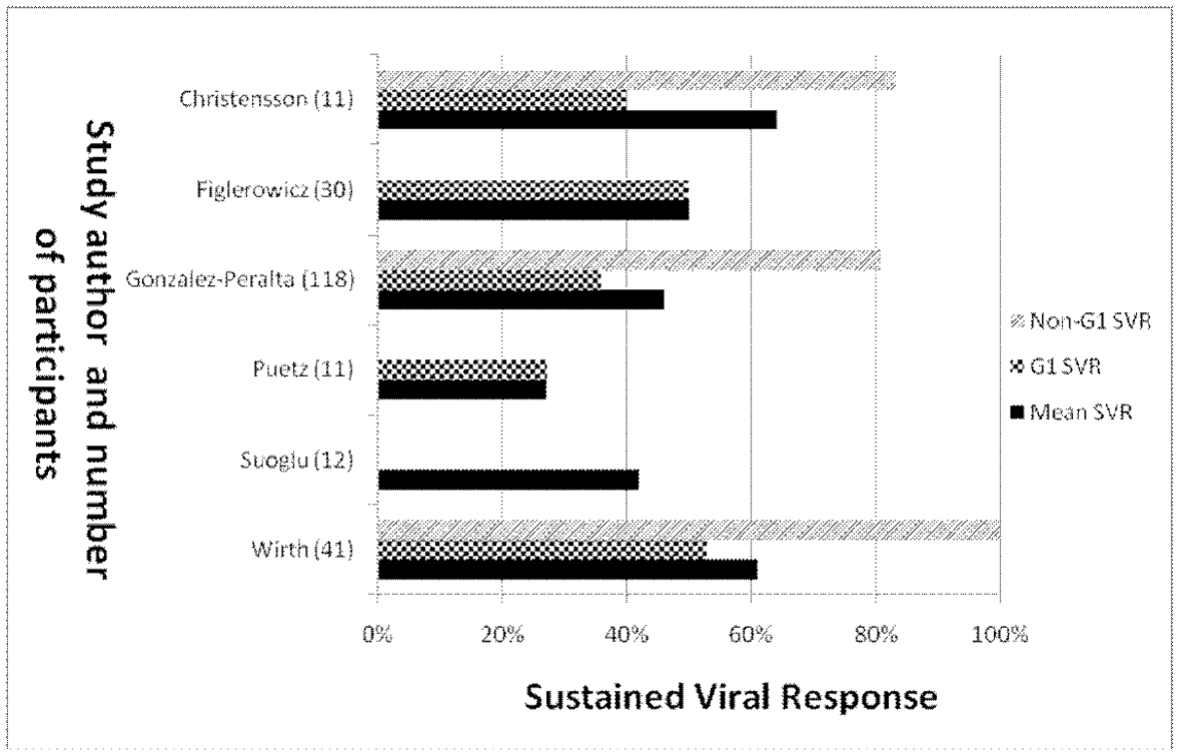
SVR by genotype of interferon/ribavirin therapy in non-randomized studies. Note the Suoglu study did not report data on genotype and all patients in the Puetz and Figlerowicz studies were infected with genotype 1.

**Figure 4:**
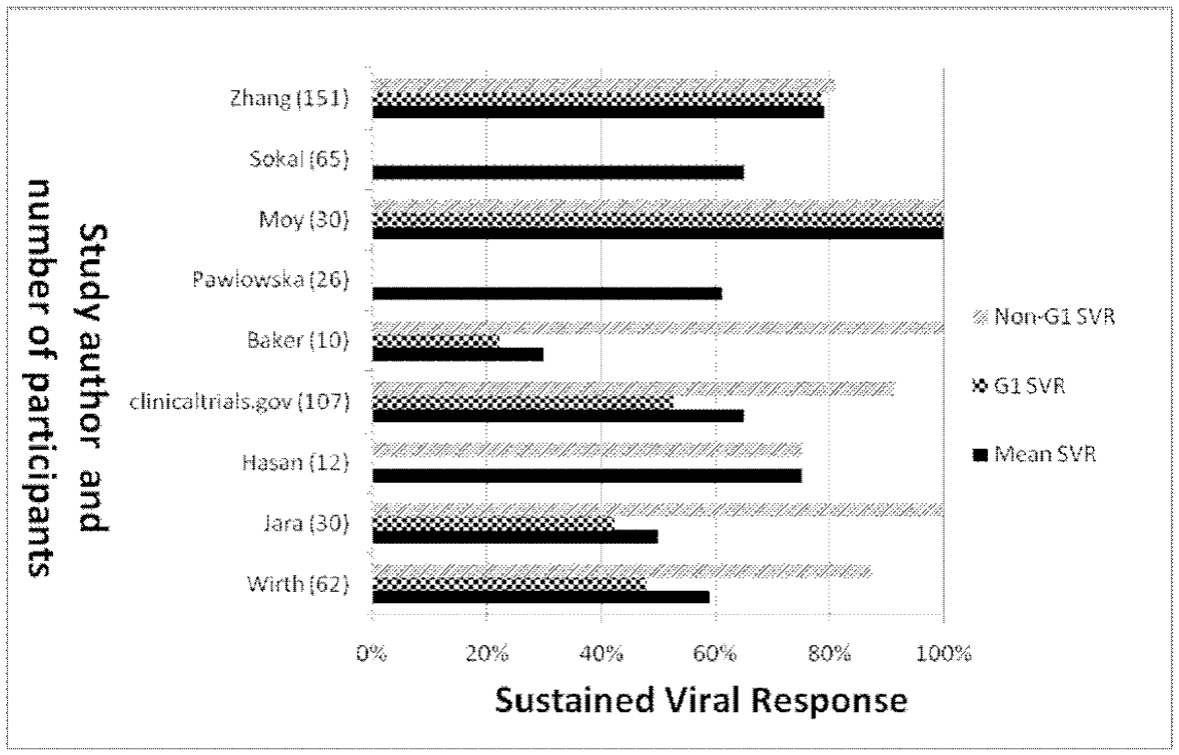
SVR by genotype of PEG-IFN/ribavirin therapy in non-randomized studies. Note all patients in the Hasan study were infected with genotype 4 and genotype 1 vs. non-genotype 1 SVR was not reported for the Pawlowska and Sokal studies.

Among the 16 interferon monotherapy therapy groups, SVR ranged from 0% to 76% (median: 37%) with 122 of 342 children (36%) achieving SVR (Figure 2). In the only study of PEG-IFN monotherapy, 6 of 14 children (43%) achieved SVR [24]. Among the 6 interferon/ribavarin combination therapy groups, SVR ranged from 27% to 64% (median: 48%) with 109 of 233 children (49%) achieving SVR (Figure 3). Among the 9 PEG-IFN/ribavarin combination therapy groups, SVR ranged from 30% to 100% (median: 63%) with 341 of 493 children (69%) achieving SVR (Figure 4).

Many of the interferon monotherapy studies did not report viral genotypes. For the interferon/ribavirin case series, the SVR for genotype 1 ranged from 27% to 53% (median: 40%). Only two patients among the interferon/ribavirin case series were reported to have genotype 4, and neither of them responded to therapy. The SVR for genotypes 2 and 3, which respond more favorably to treatment than genotypes 1 or 4, was reported in three studies as 84%, 100%, and 100%.

For the PEG-IFN/ribavirin case series where the genotype was specified, the SVR for genotype 1 ranged from 22% to 100% (median: 51%) with a combined response of 215/342 (51%) (Table S1). For genotype 2 or 3, 53 of 54 patients responded (98%) and for genotype 4, 9 of 12 patients responded in one study (75%) and 80% of patients responded in another study (number of patients not provided).

**Figure 5:**
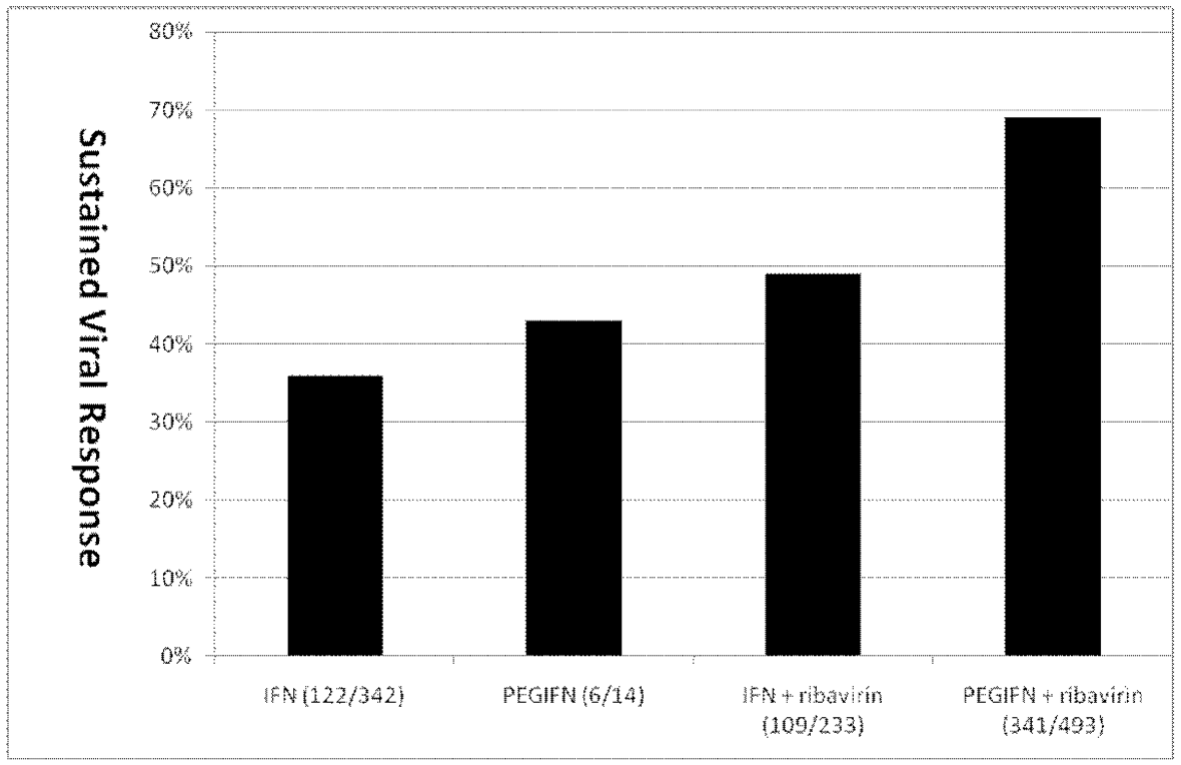
SVR and aggregate number of patients included and who achieved SVR by treatment class.

Figure 5 shows the SVR in all non-randomized and RCTs combined, showing improved results with PEG-IFN/ribavirin (statistics not applied as most studies not randomized).

## Discussion

Interferon monotherapy was the initial therapy studied for pediatric HCV infection and was the focus of 16 studies published between 1992 and 2003. Multiple dosing regimens of interferon were studied with the most common being thrice weekly interferon-alfa for a minimum of 22 weeks. A range of SVR from 0 to 76% from non-randomized studies and a SVR of 45%[18] and 69% [17] from RCTs was reported. There was only one study of PEG-IFN monotherapy with a 43% SVR [24]. Adding ribavirin to interferon was reported in 8 studies published from 2000 to 2007 with a range in SVR from 27% to 64% for the non-randomized studies and SVR of 59% for the RCT [20].These therapies are no longer used as standard therapy for adults and are unlikely to be studied further in children, especially since IFN requires thrice weekly injections versus weekly injections with PEG-IFN. Nonetheless, data on response rates is potentially useful in situations where children do not tolerate newer therapies.

The current standard of care for adults with chronic HCV is combination therapy with PEG-IFN and ribavirin [1], [15], [16]. PEG-IFN alfa-2b is approved by the US Food and Drug Administration (FDA) for children aged 3 years and older and by the European Medicines Agency with approval of PEG-IFN alfa-2a by the FDA anticipated to occur soon. In adults, a recent large multicenter RCT demonstrated no significant difference in the SVR rate between the two available peginterferon-ribavirin regimens [25].

There were 9 pediatric non-randomized studies of PEG-IFN with ribavirin with a range in SVR from 30% to 100%. The only RCT described a 53% SVR [19]. This is certainly no better in comparison to the 45% to 69% [17], [18] and 59% [20] SVR rates achieved with interferon monotherapy and combination interferon and ribavirin respectively in RCTs. However, comparison between trials is clearly flawed given differences in the populations studied and in particular the lack of comprehensive genotype-specific outcomes.

The vast majority of children will tolerate PEG-IFN and ribavirin. Although over half the children in the RCT required dose reduction of PEG-IFN, it is recognized that dose reduction was more common in adult trials than it is in clinical practice. The majority of adverse effects were mild to moderate and the rates of discontinuation of therapy were low in all groups studied.

As with adult HCV infection, genotype 1 is associated with a lower response rate to all therapies than is genotype 2 or 3 in children. In adults, response to therapy with genotype 4 is lower than with genotype 2 or 3, but there is insufficient pediatric data to be certain the same principle applies. The proportion of children with genotype 1 was 81% in the PEG-IFN ribavirin study [19] versus 70% in the interferon and ribavirin RCT [20] and an average of 54% in the two interferon monotherapy RCTs [17], [18]. The relatively low percentage of children with genotype 1 in the earlier RCTs may account for the surprisingly high SVRs. The efficacy of PEG-IFN and ribavirin in children seems comparable to adults with a SVR of 42% to 46% in adults (versus 47% in the pediatric RCT) in genotype 1 [15], [16], and 76% to 82% in genotype 2 or 3 in adults (versus 80% for non-genotype 1 in the pediatric RCT). Based on adult data, African-American ethnicity is also a risk factor for poor response [26]. Because this was recognized only recently, a minority of pediatric RCTs or case series report ethnicity.

In adults, 24 weeks of therapy for genotype 2 or 3 has been shown to be equivalent to 48 weeks [27]. Currently 48 weeks of therapy is recommended for all children in American Association for Study of Liver Disease guidelines regardless of genotype [1] but a recent small study demonstrated SVR in 16 or 18 children treated for 24 weeks with genotype 2 or 3 [28]. In adults, failure to achieve an early virologic response, as defined by at least a 2 log_10_ drop in HCV RNA at week 12 from baseline, is associated with non-response [29] and therapy is discontinued. It remains to be determined if this week 12 stopping rule applies to pediatric patients.

The current study did not address routine follow-up for children with HCV infection or when treatment of HCV should be commenced. It would seem logical to perform clinical follow-up with measurement of aminotransferases every 3 to 6 months in children who remain well. However, given the fact that about one-quarter of adults with significant fibrosis have normal aminotransferases [1], it remains very controversial if a liver biopsy should routinely be performed in children after an arbitrary time period, such as 10 years of HCV infection, even with normal aminotransferases. Transient elastography (FibroScan ®, EchoSens, Paris, France) may ultimately prove useful as a non-invasive test to identify children who are likely to have fibrosis on biopsy but this requires further study.

For genotype 1 HCV, new therapies with higher efficacy rates [30], [31]are likely to be licensed soon. We therefore would advise delaying therapy for genotype 1 HCV unless a biopsy shows significant fibrosis, or the parent or child is very insistent on proceeding. It remains controversial if patients with genotypes 2 or 3 who are likely to be compliant and to tolerate therapy should all be treated with PEG-IFN and ribavirin without a biopsy since the response rate is over 80% in adults [1] and there are no new therapies on the horizon. If the decision is made to treat children with these genotyopes, it would seem reasonable to proceed without a biopsy. For children over 2 years of age with significant fibrosis from any genotype, treatment with weight-adjusted doses of PEG-IFN and ribavirin should be offered, avoiding therapy in younger children because of the risk of neurotoxicity from interferon [32]. The decision of when to start therapy must account for the estimated or known duration of infection, genotype, degree of fibrosis (particularly in genotype 1), comorbidities, predicted compliance, expected adverse events and anticipated interference with home life, school and extra-curricular activities. Topical anesthetic creams should be considered for children with needle phobia. Despite an absence of data, we recommend using the adult early stopping rules for rapid responders and non-responders. Monitoring during therapy should follow adult guidelines [1]. These do not currently recommend routine ophthalmologic assessments. More pediatric data is required to determine if this could prevent any long-term morbidity.

Previous studies have suggested that low baseline serum HCV RNA predicts successful therapy, and that there is no correlation between response and pretreatment serum aminotransferase levels [33]. Recently, a genetic polymorphism near the *IL28B* gene on chromosome 19 has been found to be highly predictive of viral clearance with PEG-IFN and ribavirin, which explains some of the association between response rate and ethnicity [34]. A limitation of the current study is that patients could not be stratified by this polymorphism, age, ethnicity, mode of infection, pretreatment serum aminotransferase levels, pretreatment serum HCV RNA levels, or results of liver histology. Future studies should report these features for all patients in addition to genotype. It would also be useful to document if there is any risk of later relapse in children who attain a SVR.

Newer therapies are on the horizon. Phase 3 trials have been completed in adults with genotype 1 HCV using triple combination PEG-IFN, ribavirin and the protease inhibitors telapravir or boceprevir. There is a clear benefit over combination PEG-IFN and ribavirin, increasing the SVR from 38% to 41% with dual therapy up to 67% to 75% with triple therapy [30], [31]. It will be vital to collect data on the safety and efficacy of these novel combination therapies in children. Long-term follow up studies are also needed to determine the incidence and sequelae of potential neurotoxicity from PEG-INF in children and the impact of therapy on morbidity and mortality.

## Supporting Information

Table S1 (0.16 MB DOC)

Appendix S1 Search strategy.
(0.03 MB DOC)
