# Supplementary material for: Treatment of Hepatitis C in Children: A Systematic Review
Source: PLoS One. 2010 Jul 13;5(7):e11542. doi: 10.1371/journal.pone.0011542 (PMC2903479; doi:10.1371/journal.pone.0011542)
Supplement: Table S1 — (0.16 MB DOC) [file pone.0011542.s001.doc]

**Table S1. Study design and outcome of non-randomized studies of pediatric HCV therapy**

| Reference | Country | Patients | Treatment | Overall SVR | Genotype-specific SVR^1^ |
| --- | --- | --- | --- | --- | --- |
| INTERFERON MONOTHERAPY | | | | | |
| Clemente, 1994[35] | Italy | 51 | Interferon alfa-2b 3 MU/m^2^ 3 times weekly for 15 months | 21 (41%) | Not reported |
|  |  |  |  |  |  |
| Czerwionka-Szaflarska, 2000[36] | Poland | 30 | Interferon alfa ?3 MU 3 times weekly for 25 weeks | 4 (14%) at 18 months | Not reported |
|  |  |  |  |  |  |
|  |  |  |  |  |  |
| Fujisawa, 1995[37] | Japan | 18 | Interferon alfa 0.1 MU/kg daily for 2 weeks and then 3 times weekly for 22 weeks | 10 (56%) | Genotype 1: none  Genotype 2/3: 4/9 (44%)  Genotype 4: 2/2 (100%) |
| Jonas, 1998[38] | USA | 23 | Interferon alfa-2a 3 MU/m^2^ three times weekly for 12 months | 7 (30%) | Not reported |
|  |  |  |  |  |  |
| Ko, 2001[39] | Korea | 17 | Interferon alfa-2a 3 MU (<30 kg) or 6 MU (>30 kg) 3 times weekly for 6 months | 7 (41%) | Genotype 1: 1/8 (13%)  Genotype 2/3: 5/7 (71%)  Genotype 4: none |
|  |  |  |  |  |  |
| Komatsu, 1996[40] | Japan | 13 | Interferon alfa 0.1 MU/kg daily for 2 weeks and then 3 times weekly for 22 weeks | 5 (38%) | Genotype 1: none  Genotype 2/3: 1/8 (13%)  Genotype 4: none |
|  |  |  |  |  |  |
| Majda-Stanislawska, 2000[41] | Poland | 32 | Interferon alfa 3 MU (<40 kg) or 5 MU (>50 kg) three times weekly for 12 months | 5 (16%) | Not reported |
|  |  |  |  |  |  |
| Matsuoka, 1997[42] | Japan | 22 | Interferon alfa 0.1 MU/kg daily for 2 weeks and then 3 times weekly for 22 weeks | 8 (36%) | Genotype 1: none  Genotype 2/3: 7/20 (35%)  Genotype 4: 1/2 (50%) |
|  |  |  |  |  |  |
| Mozer-Lisewska, 2003[43] | Poland | 20 | Interferon alfa-2b 3 MU/m^2^ 3 times weekly for 6 months | 4 (20%) | Genotype 1: 4/19 (21%)  Genotype 2/3: 0/1 (0%)  Genotype 4: none |
|  |  |  |  |  |  |
| Nakashima, 2003[44] | Japan | 34 | Interferon alfa .1 MU/kg daily for 2 weeks and then 3 times weekly for 22 weeks | 16 (47%) | Genotype 1: 7/23 (30%)  Genotype 2/3: 8/10 (80%)  Genotype 4: none |
|  |  |  |  |  |  |
| Pensati, 1999[45] | Italy | 25 | Infereron alfa-2b 5 MU/m^2^ 3 times weekly for 12 months | 2 (8%) | Genotype 1: 1/10 (10%)  Genotype 2/3: 1/4 (25%)  Genotype 4: none |
|  |  |  |  |  |  |
| Ruiz-Moreno, 1992[46] | Spain | 12 | Interferon alfa 3 MU/m^2^ 3 times weekly for 6 months | 8 (67%) | Not reported |
|  |  |  |  |  |  |
| Sawada, 1998[47] | Japan | 26 | Interferon alfa 0.1 MU/kg daily for 2 weeks and then 3 times weekly for 22 weeks | 10 (39%) | Genotype 1: 5/12 (42%)  Genotype 2/3: 3/3 (100%)  Genotype 4: none |
| Spiliopoulou, 1999[48] | Greece | 13 | Interferon alfa-2b 3 MU 3 times weekly for 18 months | 10 (76%) | Genotype 1: 1/2 (50%)  Genotype 2/3: 2/4 (50%)  Genotype 4: 7/7 (100%) |
|  |  |  |  |  |  |
| Suoglu, 2001[23] | Turkey | 10 | Interferon alfa 3 MU/m^2^ 3 times weekly for 12 months | 3 (30%) | Not reported |
|  |  |  |  |  |  |
| Zwiener, 1996[49] | USA | 8 | Interferon alfa-2b 3 MU/m^2^ 3times weekly for 24 weeks | 0 (0%) | No patients achieved SVR |
| PEG-IFN MONOTHERAPY | | | | | |
| Schwarz, 2006[24] | USA | 14 | PEG-IFN alfa-2a 180 micrograms x BSA / 1.73 m^2^ once weekly for 48 weeks | 6 (43%) | Not reported |
| INTERFERON/RIBAVIRIN COMBINATION THERAPY | | | | | |
|  |  |  |  |  |  |
| Christensson, 2000[50] | Sweden | 11 | Interferon alfa 5 MU 3 times weekly + 15 mg/kg/day ribavirin for 48 weeks | 7 (50%) | Genotype 1: 2/5 (40%)  Genotype 2/3: 5/5 (100%)  Genotype 4: 0/1 (0%) |
|  |  |  |  |  |  |
| Figlerowicz, 2004[51] | Poland | 30 | Interferon alfa-2b 3 MU 3 times weekly + 15 mg/kg/day ribavirin for 48 weeks | 15 (50%) | All patients were genotype 1 |
|  |  |  |  |  |  |
| Gonzalez-Peralta, 2005[52] | Multiple countries | 118 | Interferon alfa-2b 3 MU/m^2^ 3times weekly + 15 mg/kg/day ribavirin for 48 weeks | 54 (46%) | Genotype 1: 33/92 (36%)  Genotype 2/3: 212/25 (84%)  Genotype 4: 0/1 (0%) |
|  |  |  |  |  |  |
| Puetz, 2004[53] | USA | 11 | Interferon alfa 3 MU 3 times weekly + 1000 mg/day ribavirin for 12 months | 3 (27%) | All patients were genotype 1 |
| Suoglu, 2001[23] | Turkey | 12 | Interferon alfa, 3 MU/m^2^ 3 times weekly + 15 mg/kg ribavirin for 12 months | 5 (42%) | Not reported |
| Wirth, 2002[54] | Germany | 41 | Interferon alfa-2b 3 MU/m^2^ or 5 MU/m^2^ 3 times weekly + ribavirin 15 mg/kg/day for 12 months | 25 (61%) | Genotype 1: 18/34 (53%)  Genotype 2/3: 7/7 (100%)  Genotype 4: none |
| PEG-IFN/INTERFERON COMBINATION THERAPY | | | | | |
| Baker, 2007[55] | USA | 10 | PEG-IFN alfa-2b 1.5 micrograms/kg once weekly + 800 mg/day ribavirin for 48 weeks | 3 (30%) | Genotype 1: 2/9 (22%)  Genotype 2/3: 1/1 (100%)  Genotype 4: none |
|  |  |  |  |  |  |
| Jara, 2008[56] | Spain | 30 | PEG-IFN alfa-2b 1.0 micrograms/kg/week + 15 mg/kg/day ribavirin for 28 to 48 weeks | 15 (50%) | Genotype 1: 12/27 (44%)  Genotype 2/3: 3/3 (100%)  Genotype 4: none |
|  |  |  |  |  |  |
| Wirth, 2005[57] | Germany | 62 | PEG-IFN alfa-2b 1.5 micrograms/kg/week + 15 mg/kg/day ribavirin for 48 weeks | 36 (59%) | Genotype 1: 22/46 (48%)  Genotype 2/3: 13/13 (100%)  Genotype 4: 1/2 (50%) |
| Hasan, 2006[58] | Not reported | 12 | PEG-IFN alfa-2b 1.5 micrograms/kg/week for 48 weeks | 9 (75%) | All patients were genotype 4 |
|  |  |  |  |  |  |
| clinicaltrials.gov, 2008[59] | Multiple countries | 107 | PEG-IFN alfa-2b 60 micrograms/m^2^ once weekly + 15 mg/kg/day ribavirin for 48 weeks | 70 (65%) | Genotype 1: 38/72 (53%)  Genotype 2/3: 93% ^2^  Genotype 4: 80%^2^ |
| Pawlowska, 2010 [60] | Poland | 26 | PEG-IFN alfa-2a + ribavirin (dose not specified) | 16 (61%) | Genotype specific responses were not reported |
| Moy, 2010 [61] | USA | 30 | PEG-IFN alfa-2a + ribavirin (dose not specified) | 30 (100%) | Genotype 1: 13/13 (100%)  Genotype 2/3 10/10 (100%)  Genotype 4: 1/1 (100%) |
| Sokal, 2009 [28] | 5 in Europe | 65 | PEGIFN alfa-2a 100 micrograms/m^2^ once weekly + ribavirin 15 mg/kg/day for 24 weeks (genotypes 2/3) or 48 weeks (other genotypes) | 43 (65%) | Genotype 1/4/5/6: 27/47 (57%)  Genotype 2/3: 16/18 (89%) |
|  |  |  |  |  |  |
| Zhang, 2009 [62] | China | 151 | PEGIFN alfa-2a 104 micrograms/m^2^ or PEGIFN alfa-2b 1.5micrograms/kg once weekly + ribavirin 15-20 mg/kg/day for 48-72 weeks | 119 (79%) | Genotype 1: 102/130 (78%)  Genotype 2: 9/10 (90%)  Others genotypes: 8/11: 73% |

All studies were uncontrolled before-after studies except for Suoglu, 2001. If the type of interferon alfa is not specified, it is because the study did not provide this information.

^1^Reported as ‘none’ if no patients were infected with that particular genotype or if patients infected with that particular genotype were also infected with another genotype. Patients with mixed genotypes were excluded from this column so the number of patients is lower than in the previous column.

^2^The number of patients was not reported for genotypes 2, 3, or 4.
