# Supplementary material for: Treatment of Hepatitis C in Children: A Systematic Review
Source: PLoS One. 2010 Jul 13;5(7):e11542. doi: 10.1371/journal.pone.0011542 (PMC2903479; doi:10.1371/journal.pone.0011542)
Supplement: Appendix S1 — Search strategy. (0.03 MB DOC) [file pone.0011542.s002.doc]

**Appendix S1: Ovid MEDLINE® (1950 to April Week 2 2009)**

1. exp Hepatitis C/dh, de, tu, th, dt, pc, rh

2. exp Hepatitis C/

3. (hepatitis adj c).ti,ab.

4. (hepacvirus or hepatitis-c or HCV).ti,ab.

5. ("chronic hepatitis" adj5 c).ti,ab.

6. or/2-5

7. exp complementary therapies/ or exp drug therapy/ or exp electric stimulation therapy/ or exp emergency treatment/ or exp Treatment Outcome/

8. 6 and 7

9. 1 or 8

10. exp Infant/

11. (Infant* or infancy or Newborn* or Baby* or Babies or Neonat* or Preterm*).mp.

12. exp Child/

13. (Child* or Schoolchild* or School age* or Preschool* or Kid or kids or Toddler*).mp.

14. exp Adolescent/

15. Adoles*.ti,ab.

16. (Teen* or Boy* or Girl*).mp.

17. exp Minors/

18. minors*.mp.

19. exp Puberty/

20. (Pubert* or Pubescen* or Prepubescen*).mp.

21. exp Pediatrics/

22. (Pediatric* or Paediatric* or Peadiatric*).mp.

23. exp Schools/

24. (Nursery school* or Kindergar* or Primary school* or Secondary school* or Elementary school* or High school* or Highschool*).mp.

25. or/10-24

26. exp Adolescent/ and exp Adult/

27. or/10-13,15-24

28. 26 not 27

29. 25 not 28

30. 9 and 29

31. randomized controlled trial.pt.

32. clinical trial.pt.

33. randomi?ed.ti,ab.

34. placebo.ti,ab.

35. dt.fs.

36. randomly.ti,ab.

37. trial.ti,ab.

38. groups.ti,ab.

39. or/31-38

40. animals/

41. humans/
